# Ruptured Parasitic Dermoid Cyst in Blunt Abdominal Trauma

**DOI:** 10.24248/eahrj.v8i2.776

**Published:** 2024-06-26

**Authors:** Emanuel Q. Nuwass, Martini Gemuwang, Hayte M. Samo, Daudi Lotto, Fides Canuty

**Affiliations:** a Department of General Surgery, Haydom Lutheran Hospital, Manyara, The United Republic of Tanzania; b Department of Radiology, Haydom Lutheran Hospital, Manyara, The United Republic of Tanzania

## Abstract

Ruptured parasitic dermoid cyst is one of the rare conditions that results from auto-amputation and re-implantation following torsion from the ovary and omentum, among other sites. Due to trauma to the abdomen, it may rupture, resulting in spillage of its contents and causing chemical peritonitis. The diagnosis is based on clinical presentation complimented by abdominal ultrasound in low resource settings. A case of a 44-year-old (Iraqw by tribe) who presented with clinical features of acute generalized abdominal pain and distension for two days following blunt abdominal trauma. The abdominal ultrasound revealed a mass of mixed echogenicity with intraperitoneal free fluid. She underwent laparotomy, revealing ruptured parasitic dermoid cyst adhered to the anterior abdominal wall and urinary bladder, then excised. The high degree of suspicion of ruptured parasitic dermoid cyst is justified in adult patient with chemical peritonitis manifestation. The laparotomy is surgical management of choice in low-resource settings followed by prolonged follow-up.

## BACKGROUND

A mature cystic teratoma is synonymously called a dermoid cyst. It arises from three germ cells, which include endoderm (mucinous, ciliated epithelium of gastrointestinal, thyroid, and bronchial tissues), ectoderm (skin, brain), and mesoderm (teeth, cartilage, bones, hairs, and fat). These constitute around 30% of all ovarian tumors. ^1^ It is commonly located in the ovary, caecum, and pelvis and is rarely found in the retroperitoneal space.^2,3,4^

Furthermore, dermoid cystic teratoma can present in a rare form called parasitic dermoid cysts, with an incidence of around 0.4% of all ovarian dermoid cysts. The cause is not yet certain; however, some postulate that the cause can be due to the auto-amputation and re-implantation of a dermoid cyst from a distant common site of origin like the ovaries as a result of torsion and hence detachment. This is followed by migration and adherence to a new site, resulting in neovascularization.^1^ The common sites for parasitic dermoid cysts are reported to be the pouch of Douglas, omentum, and inguinal canal, as recently reported in the reflection of the uterus at the anterior abdominal wall.^5,6,7^ Clinically, most of these cases will present with a history of abdominal pain for at least a few years that may later result in a torsion.^8^

The clinical manifestation of parasitic dermoid cysts is determined by the site, size, and complications they present with, as well as other conditions accompanied by, for instance, pregnancy. The parasitic dermoid cyst may be found as an incidental finding and sometimes may present with overt clinical symptoms. The presentation commonly results from complications like torsion, rupture, haemorrhage, and infection. The common clinical presentation of parasitic dermoid cysts includes lower abdominal pain, abdominal distension, palpable mass, pain, or vomiting.^1,9^ The ruptured parasitic dermoid cyst is a rare complication and commonly occurs spontaneously. In rare cases, it may be caused by pressure due to pregnancy, torsion, infection, or malignant transformation during surgical procedures, and in a few cases, it may be caused by trauma due to the patient falling. Other causes of rupture include motor traffic accidents and vigorous exercise.^10,11^ A ruptured dermoid causes spillage of its fluid and other contents, which may lead to chemical peritonitis and later granuloma formation. Chemical peritonitis is a surgical emergency that requires immediate intervention and is a life-threatening complication requiring prompt diagnosis and management.^1,12^ The diagnosis of a ruptured dermoid cyst is clinically based on typical features of peritonitis and radiological imaging findings. The radiological imaging tests include computed tomography, which has a higher yield of up to 88%; magnetic resonance imaging, which has a low yield of around 50%; abdominal ultrasound, which has a low yield of around 49%; and x-rays, which have the lowest diagnostic power. The management of ruptured dermoid cysts includes prompt laparoscopy and laparotomy for immediate excision of the ruptured teratoma and drainage of its spilled contents, along with antibiotic administration and fluid resuscitation. In complicated cases, those with adhesions may require a laparotomy.^11,12^ Here we report a rare case of a ruptured parasitic dermoid cyst presenting with chemical peritonitis following a blunt abdominal trauma to share the challenge of its diagnosis and management in low-resource settings.

## CASE PRESENTATION

A 44-year-old female (Iraqw by tribe) from Mbulu who presented in the emergency unit at Haydom Lutheran Hospital with a chief complaint of acute generalised abdominal pain for two days that occurred following falling over a blunt object, and she sustained blunt abdominal trauma. The abdominal pain was associated with abdominal distension and episodes of bloody urine without any active bleeding; however, she denies a history of vomiting, constipation, and fever. She reports a history of progressive awareness of heartbeat in the course of an illness associated with generalised body weakness without headache over a few days. Before the event of trauma, she reported a longstanding history of on-and-off moderate lower abdominal pain for 9 years associated with episodes of difficulty passing urine, denying any events of per-vaginal bleeding and discharge without history of back pain.

Her family and social history were that she was a housewife, married, living with her husband, and working as a peasant. She had five children who were born at the district hospital, where the third was delivered by caesarean section and the rest were born by vaginal delivery without any complications, and the children are alive and well. She denied any family history of this kind of illness or any other chronic illness.

During the general examination, it was found that she was sick-looking, weak, mildly pale, and had tachycardia with stable blood pressure and oxygen saturation. On abdominal examination, the findings were a symmetrically distended abdomen, a sub-umbilical midline incision (SUMI) scar due to Caesarean section, mild tenderness without guarding or rebound tenderness, and positive shifting dullness but no ballotable organ or mass.

**Figure 1.**
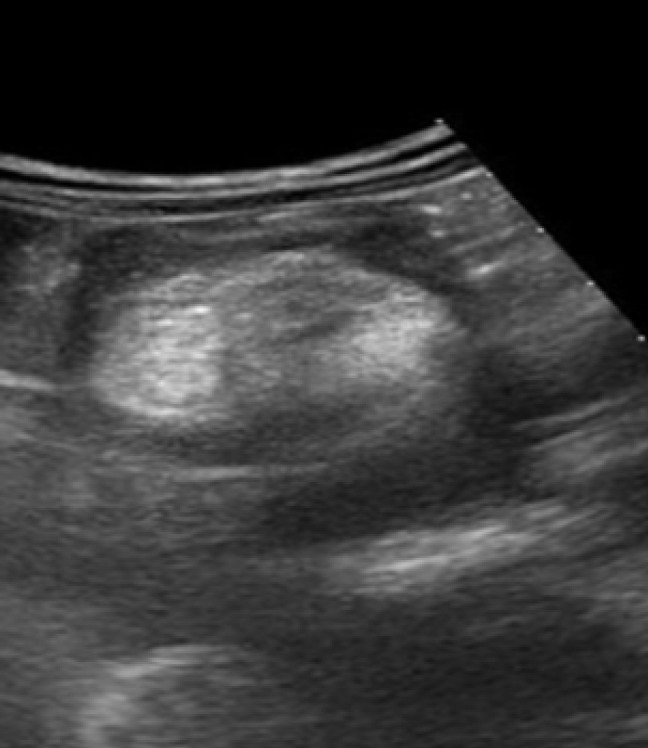
An Ultrasound Picture Showing a Mixed Echogenic (Hyperechoic with Relative Acoustic Shadowing and Hypoechoic) Mass

The laboratory test and full blood picture revealed a slightly elevated platelet count of 620/Ul; otherwise, all other tests were in the normal range, including liver and renal function tests. The abdominal ultrasound findings were a mass of mixed echogenicity (Figure 1) noted in the pelvic anterior superior to the urinary bladder, measuring 7.6 cm in width by 4.5 cm in depth and 5.5 cm in length, surrounded by fluid, and a significant amount of clear fluid in the Morrisons, splenorenal, and pelvic fluid with normal intra-abdominal organs. The plain abdominal X-ray was normal.

Based on these findings, a diagnosis of blunt abdominal trauma with suspicion of visceral injury and intraabdominal mass was made, and an emergency explorative laparotomy was planned on the third day. A signed consent form was obtained from the patient. The blood workouts and other preoperative preparations were completed.

**Figure 2.**
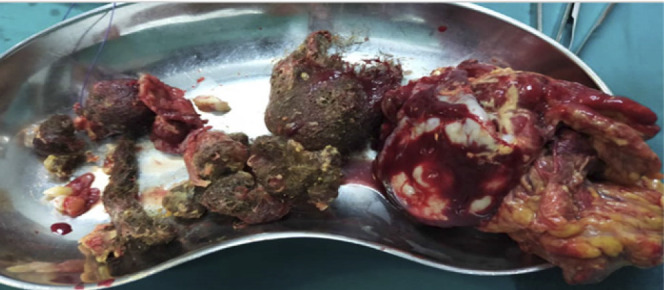
Content of the Parasitic Dermoid Cyst After Removal From the Anterior Abdominal Wall

Treatment: an explorative laparotomy was done on the third day post-admission, and the intraoperative findings were strawberry intraperitoneal fluid, a hard irregular semi-mobile mass that was likely part of a ruptured cyst. The mass had bowels, and the omentum adhered over them. This mass was found in the pelvic cavity, adhering to the anterior abdominal wall and the urinary bladder. The mass was completely resected, and its contents were hair bundles, teeth, and other unidentifiable structures (Figure 2), and sent for histopathology. The intraoperative diagnosis of an incidental parasitic mixed matured teratoma (Parasitic Dermoid cyst) that ruptured due to trauma was reached.

Outcome and follow-up: The postoperative care was uneventful, and the patient was discharged on day seven without any complications. The histopathology finding of the mass was a cystic structure without epithelial lining but having a heavy mixed inflammatory cell suggestive of a ruptured pseudocyst with peritonitis.

The postoperative follow-up was done as an outpatient for six months with good progress and was planned to be further followed up for up to two years.

## DISCUSSION

Mature cystic teratoma is among the most common ovarian tumours, constituting around 5–30% of all ovarian cysts. It is common among women aged 20–30, and some cases are seen in older ages as well. This case of the parasitic dermoid cyst in the anterior abdominal wall is a rare presentation and constitutes around 0.4% incidence, and its occurrence results from a torsion of the dermoid cyst through autoamputation and reimplantation, which occurs in 16.1% of all cases.^1,5,8^

This case presented with a history of abdominal pain and distention with mild tenderness, which are similar to the presentation in cases of chemical peritonitis as retrieved in case reports of traumatic rupture of the dermoid cyst; however, most of the cases are asymptomatic.

This case further presented a short history of blunt abdominal trauma, which is likely an explanation of the cause of the rupture of the dermoid cyst as reported in similar studies.^13^ Other causes include spontaneous rupture, pressure due to pregnancy, torsion, infection, and malignant transformation during surgical procedures.^10,11^

The intraoperative findings of a free uterus and ovaries and also the presence of longstanding abdominal pain for nine years could explain that this dermoid cyst auto-amputated and migrated then re-implanted into the anterior abdominal wall in the reflection of ureterovesical membrane and hence attaching to the anterior abdominal wall with development of neovascularization then then adhering to the urinary bladder wall and abdominal wall. This process explains the postulation of the cause of parasitic dermoid cystic teratoma as reported in various studies.7, 8, and 15: Literature reports implantation into other sites like the pouch of Douglass and omentum. ^14^ The history of longstanding abdominal pain for nine years could explain the auto-amputation process and reimplantation, where the main reason could be torsion.

The clinical diagnosis was challenging in this case due to a mild form of peritonitis; however, the diagnosis of abdominal visceral injury was reached due to fluid collection found by abdominal ultrasound. Conservative management initiated included intravenous antibiotics and intravenous fluids while closely observing the patient's condition.

The radiologic investigation done included the x-ray abdomen, which had normal findings, and abdominal ultrasound findings, which showed significant peritoneal fluid with a mass of mixed echogenicity surrounded by fluid. In radiologic diagnosis for parasitic dermoid cysts, computer tomography and magnetic resonance are superior due to their sensitivity to fat in the cyst.^1^ The patient could have benefited from computer tomography as it has a good yield; however, abdominal ultrasound is reported to be adequate to diagnose dermoid cysts in the intraabdominal cavity^2^ and assist in decisions, especially in a low-resource setting.

The mode of treatment includes emergency explorative laparotomy with complete excision of the ruptured dermoid cyst from the anterior abdominal wall, while another surgical option in higher centres includes laparoscopic removal, as reported with an even better surgical outcome.^2,15^

Adequate irrigation of the pelvic cavity is required to ensure the removal of spilled fluid and associated cyst content required to be done.^14^ Postoperatively, antibiotics and fluid resuscitation according to local recommendations are key to optimising the postoperative outcome. The histological evaluation of tissue is important to determine the diagnosis and the longer-term postoperative outcomes. On discharge, prolonged follow-up plans are key due to anticipated late complications like peritonitis, abscess, bowel obstruction, and an increased risk of malignancy.^11^

## CONCLUSION

The mature mixed teratoma can present in adulthood with various presentations, including ruptured parasitic dermoid cysts and chemical peritonitis due to trauma or any other cause. The incidental finding of the ruptured dermoid cyst should be highly suspected as the cause of chemical peritonitis. A timely clinical diagnosis supported by abdominal ultrasound findings and prompt laparotomy is important to prevent associated complications, followed by prolonged postoperative follow-up in low-resource settings.

Written informed consent was obtained from the patient for the publication of this case report and accompanying images.
